# Clarifying SABA overuse: Translating Canadian Thoracic Society guidelines into clinical practice

**DOI:** 10.1186/s13223-022-00690-2

**Published:** 2022-06-11

**Authors:** Anne K. Ellis, Vanessa Foran, Alan Kaplan, Patrick D. Mitchell

**Affiliations:** 1 Division of Allergy & Immunology, Department of Medicine, Queen’s University, 76 Stuart St, Kingston, ON K7L 2V7 Canada; 2 Asthma Canada, Toronto, ON Canada; 3 York Region, and Respiratory Effectiveness Group, York region, ON Canada; 4 School of Medicine, Trinity College, Dublin, Ireland

**Keywords:** Asthma, Controlled, Rescue, Short-acting beta agonist, Overreliance, Overuse, Uncontrolled

## Abstract

Patients with asthma frequently over rely on short-acting beta-agonists (SABA) to treat acute symptoms. This can adversely impact quality of life and increase the risk of exacerbations. SABA overuse is also associated with an increased risk of mortality. In their 2021 update on the diagnosis and management of mild asthma, the Canadian Thoracic Society (CTS) newly recommended that a combination inhaled corticosteroid (ICS) and long-acting beta-agonist, specifically budesonide/formoterol, may be used as-needed (PRN) as an alternative reliever to SABA. The CTS developed an algorithm as a guide for deciding for whom PRN budesonide/formoterol versus PRN SABA is appropriate as a reliever. While the CTS algorithm provides necessary and precise guidance, the somewhat complicated requirements for determining control and exacerbation risk may still end up leaving some patients at-risk of SABA overreliance. This communication simplifies the reliever decision algorithm developed by the CTS for application in daily practice. A 30-s evaluation of 2 simple questions related to reliever use can usually accurately assess if a patient’s asthma is controlled: How many SABA canisters do you use a year AND how many times do you use SABA a week? If the patient indicates use of > 2 SABA canisters per year or > 2 administrations of SABA per week for any reason, the patient does not have controlled asthma and PRN SABA is not an appropriate treatment regimen. Similarly, for patients using PRN ICS/formoterol, more than 2 administrations per week indicates a clinical review and reevaluation of their management, including augmentation. An education process is essential to inform patients, caregivers, and healthcare providers that overuse of any reliever is not acceptable and is potentially harmful.

## Introduction

Short-acting beta-agonists (SABA) have long been regarded as essential as a reliever medication to treat acute symptoms of asthma and to prevent symptoms in response to known triggers (e.g., exercise or allergen exposure). However, overuse of SABA is a powerful indicator of poor asthma control. Patients and some healthcare professionals frequently believe that asthma symptoms and the need for frequent SABA use are simply a fact of life in living with asthma (Box 1).

Patients are given SABA early in the course of asthma management, often even before undergoing lung function testing. As-needed (PRN) SABA was recommended as the first step of management in older asthma treatment guidelines [1]. Thus, from the very beginning of their asthma management, patients came to believe that treating symptoms alone was acceptable [2]. In addition, the effects of SABA are felt immediately, giving patients the perception that it is helping control their asthma. As a result, SABA becomes viewed by patients as a “quick fix” compared with no immediate perceptible relief from inhaled corticosteroid (ICS) control [3]. When patients believe that their symptoms are being controlled by frequent SABA use, they negate the importance of concomitant ICS-containing controller use, leading to intentional or non-intentional non-adherence to controller ICS or denial of the need for it [2, 4]. Such situations can be dangerous since studies have demonstrated a clear relationship between high-dose or frequent (i.e., ≥ 3 canisters per year) SABA use and a deterioration of asthma control, risk of exacerbations, and mortality. [5–7]

**Fig. 1:**
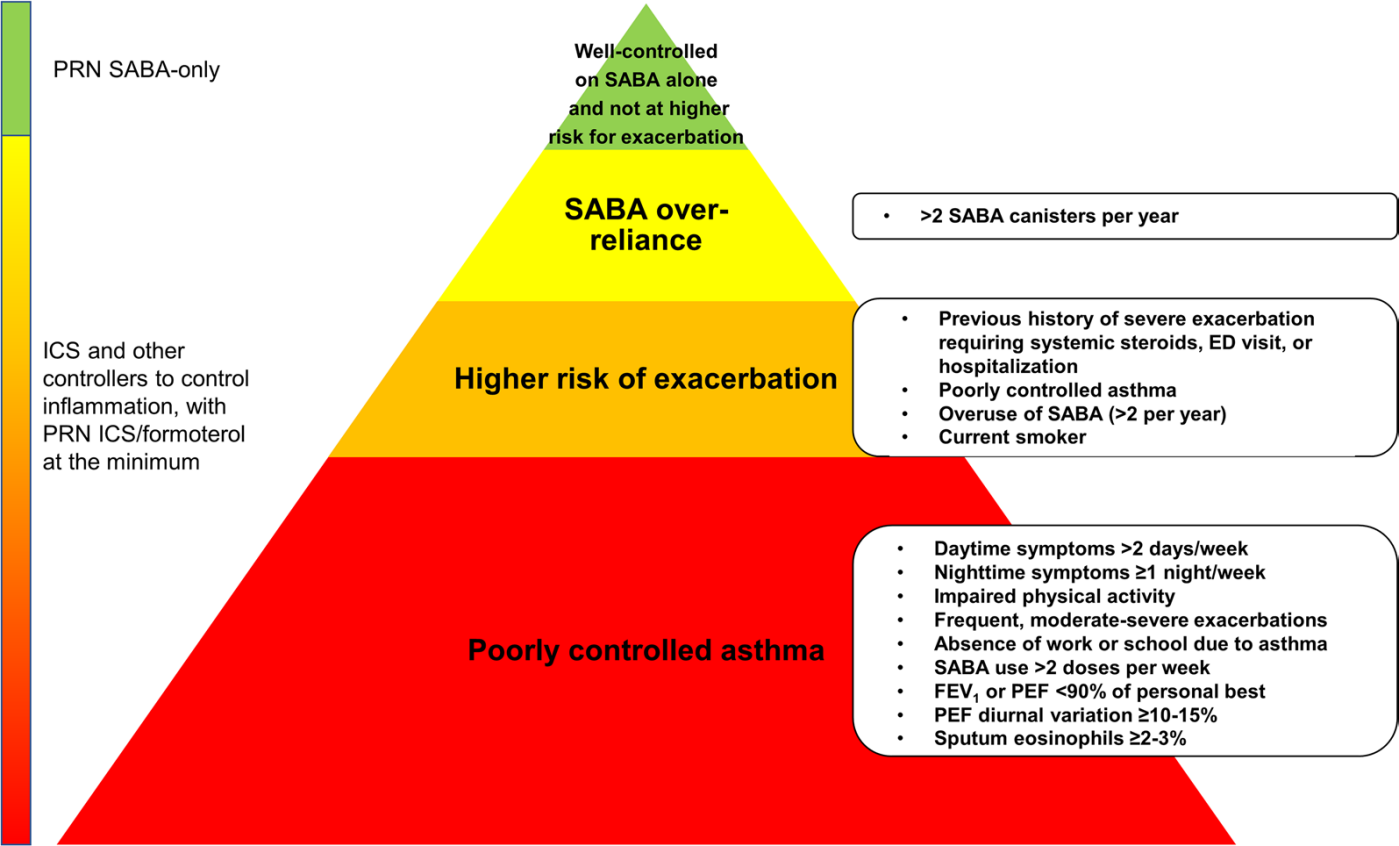
Reliever selection for patients with very mild or mild asthma. Higher exacerbation risk and poorly controlled asthma as defined by the Canadian Thoracic Society [9]. As-needed (PRN) short-acting beta-agonist (SABA) should be reserved only for patients with very mild or mild asthma who are not over-reliant on SABA, whose asthma is well-controlled on SABA alone, and who are not at higher risk for exacerbation. ED, emergency department; *FEV*_*1*_ forced expiratory volume in 1 s, *ICS* inhaled corticosteroid, *PEF* peak expiratory flow

The Global Initiative for Asthma has evolved its treatment paradigm so that low dose ICS/formoterol is now the preferred reliever approach for adults and adolescents, even at the mildest asthma severity [8]. PRN SABA is reserved for those who are on a controller medication, without exacerbations, and for whom ICS/formoterol is not possible or not preferred [8]. In their 2021 update on the diagnosis and management of very mild to mild asthma, the Canadian Thoracic Society (CTS) recommended that a combination ICS and long-acting beta-agonist (LABA), specifically budesonide/formoterol, may be used PRN as an alternative reliever to SABA [9]. The use of a PRN ICS plus a fast-acting LABA is a win–win situation, providing quick relief (within 5 min) that the patient can sense, while administering at least some degree of controller ICS medication. PRN budesonide/formoterol is approved as a reliever medication for ages ≥ 12 years in Canada. An algorithm was developed by the CTS as a guide for deciding for whom PRN budesonide/formoterol versus PRN SABA as monotherapy in very mild asthma would be appropriate as a reliever [9]. The algorithm suggests that adults and adolescents who are well controlled on PRN SABA or on no medication (e.g., very mild asthma) who are not at higher risk of exacerbations can continue on PRN SABA. “Higher risk” for exacerbations is defined as having any of the following: any history of a previous severe asthma exacerbation requiring systemic steroids, an emergency department visit, or hospitalization; poorly controlled asthma per CTS criteria; use of more than 2 SABA inhalers a year, or current smoker (Fig. 1). The definition of “well controlled” by CTS criteria is detailed and has some caveats (Fig. 1) [9]. While the CTS algorithm provides necessary and precise guidance, the somewhat complicated requirements for determining control and exacerbation risk may still end up leaving some patients at-risk to over rely on SABA if the patient overestimates their asthma control or simply ignores worsening symptoms. Furthermore, patients may not understand that more severe asthma may appear as episodic when in actuality it is not. Therefore, the goal of this communication is to simplify the reliever decision algorithm developed by the CTS for application in daily practice.

Box 1. Patient experience at age 26 years *I was first diagnosed with asthma as a baby and had severe asthma for my entire childhood and was hospitalized too many times to count. As a child, I was on multiple types of asthma medications and using a nebulizer every few hours on a daily basis for years. It wasn’t until I became a teenager that my asthma became properly controlled through a combination of medications and exercise.*

My asthma was well controlled throughout high school. I got in shape and was less reliant on my inhalers to manage my asthma. It wasn’t until I moved out on my own for university that my asthma became dangerously uncontrolled. Over a five-year period,* my asthma became progressively worse. I was relying solely on my rescue inhaler to manage my asthma, taking it up to 15 + times per day. I would wake up multiple times throughout the night needing to take my blue puffer. I had frequent asthma flare ups, was unable to be as active as I desired, and was always stressed about when the next asthma attack would happen.

*Despite all these clear signs of uncontrolled asthma, I shrugged it off and continued to go through blue puffer after blue puffer. This pattern of poor asthma control continued with my breathing spiraling out of control until it culminated in a major asthma attack where I nearly lost my life. I didn’t have my rescue inhaler on hand in the situation and had to rush to get to it in time. When I did reach it, I distinctly recall laying on the floor taking the blue puffer over and over again, soaked in sweat and desperately fighting for breath.*

*Once I decided to get my asthma under control my whole quality of life was improved. I saw my doctor and was put on a controller medication (an ICS/LABA), which I take twice daily. Now my asthma is completely controlled. I live completely symptom-free, sleep through the night without needing to take my medication, and rarely, if ever, use my rescue inhaler, but I do make sure to always carry it with me in case of an emergency. Just following a proper treatment plan can make such a tremendous difference and transform a patient’s quality of life.*

*I, like so many Canadians living with asthma, thought “it’s just asthma” and that living with frequent symptoms and anxiety were just part of the condition. Now I know that it’s never “just asthma” and that with a controller medication it’s fully possible to live a symptom-free life and not be held back by your asthma from being active and living life to the fullest.*

*My journey from overreliance on my rescue inhaler with chronic symptoms to a fully symptom free life thanks to a controller medication exemplifies the incredible difference that properly managing your asthma and following a treatment plan can have.*

*During this period the patient was using walk-in clinics for healthcare, with no family care physician or specialist to oversee his asthma management.

## Simplified evaluation of SABA overreliance

Two SABA puffs twice-weekly equates to 208 doses per year, and each SABA canister has 200 doses. A 30-s evaluation of 2 simple questions can usually accurately assess if a patient’s asthma is controlled:

1. How many SABA canisters do you use a year?
2. How many times do you use SABA a week?

**Fig. 2:**
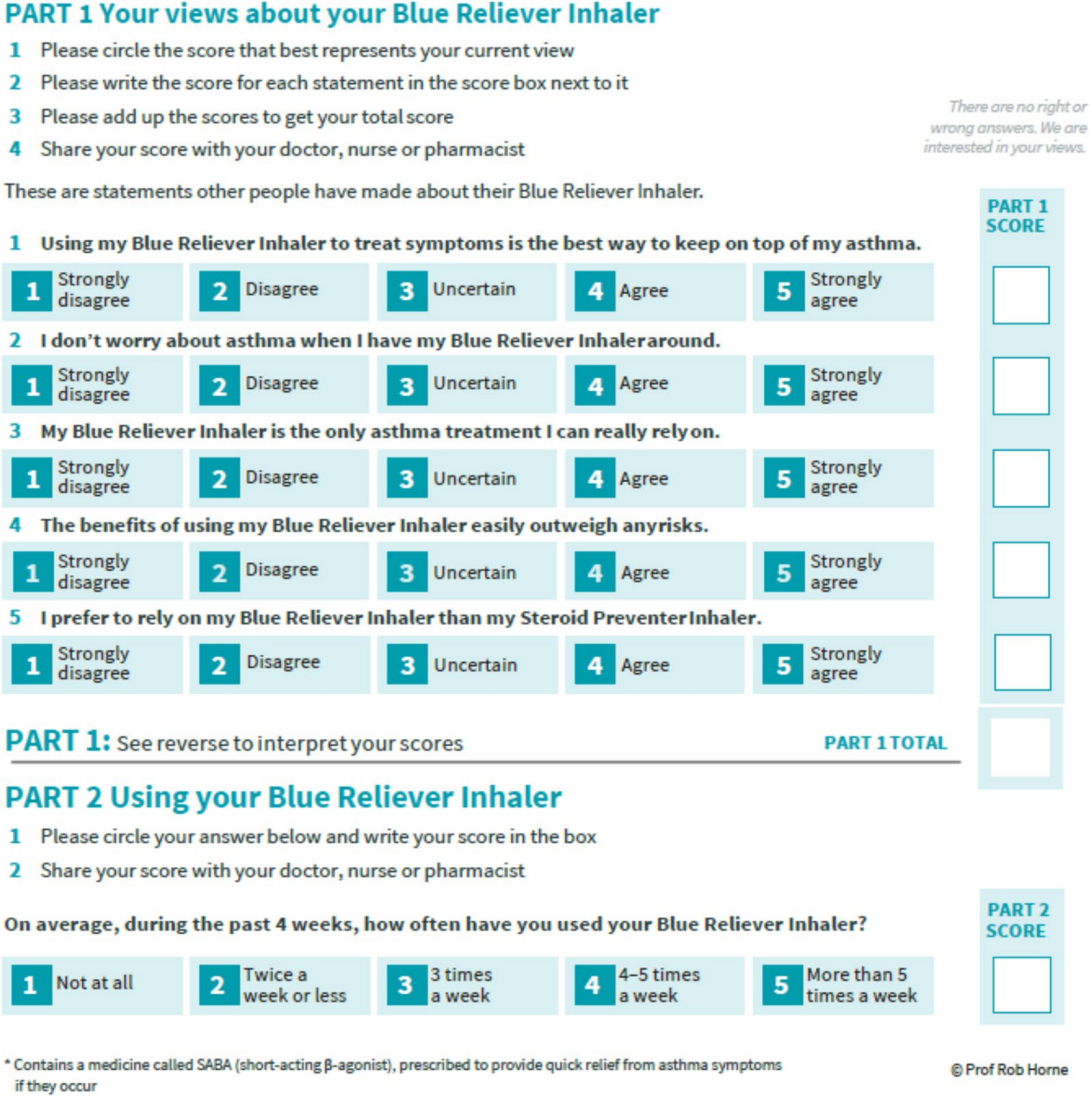
Reliever reliance test. [11] Reproduced with permission from Prof. Rob Horne.

If the patient indicates use of > 2 SABA canisters per year or > 2 administrations of SABA per week, for any reason including prevention of exercise-induced asthma or in anticipation of allergen-induced symptoms, the patient does not have controlled asthma. Those individuals who have controlled asthma based on the responses to the 2 questions above and who are at low risk of exacerbations are the individuals for whom PRN SABA is still adequate, assuming they are adherent to their controller therapies when prescribed. On the other hand, those individuals whose responses indicate their asthma is uncontrolled should be prescribed, at minimum, a daily ICS with PRN SABA, or for those over 12 years PRN budesonide/formoterol could be an alternative, as a starting point. The CTS actually recommends ICS plus PRN SABA over PRN ICS/formoterol based on results from the SYGMA1 study, which demonstrated that ICS plus PRN SABA compared with PRN ICS/formoterol resulted in significantly more well-controlled asthma weeks (44.4% vs 34.4%, respectively) and a similar annualized severe exacerbation rate (0.09 vs 0.07, respectively) [9, 10]. The exception is if poor adherence is considered, in which case PRN ICS/formoterol is preferred [9]. Incorporating a corticosteroid-containing therapy at the beginning of asthma treatment could change patient relationships with their SABA. Encouraging regular ICS use or beginning with ICS are both options. Irrespective of the new regimen selected (daily ICS with PRN SABA or PRN ICS/formoterol), making this change to allow for protection against overreliance on SABA and achieve well controlled asthma can be life-changing for patients (Box 1). Having a patient complete the Reliever Reliance Test may provide a better understanding of why a patient is over-reliant on SABA (Fig. 2) and give the clinician an opportunity to meaningfully discuss the issue with the patient. [11]

Two studies, one randomized double-blind [10] and one randomized open-label [12], have demonstrated a decrease in the rate of severe exacerbations when low dose ICS/formoterol is used as a reliever medication compared with PRN SABA alone. Thus, PRN budesonide/formoterol is a superior and safer option than PRN SABA for most adult and adolescent patients using excessive SABA therapy, who have very mild to mild asthma, and who are not on a daily controller medication. However, stepping up to PRN ICS/formoterol from PRN SABA may still not be sufficient to provide adequate control. Patients who are using PRN ICS/formoterol should also be asked how many times they use this form of medication a week as a reliever. More than 2 administrations per week should mandate a clinical review, with verification of the asthma diagnosis to rule out vocal cord disorder or obesity-related dyspnea and reevaluation of their asthma management strategy, including treatment augmentation to a regular controller therapy.

## Conclusions

Excessive reliance on SABA therapy is potentially harmful for patients with asthma, adversely affecting their quality of life and increasing their risk of exacerbations and mortality. Using the reliever decision algorithm developed by the CTS or the simplified approach we propose, very few patients will actually fit into the PRN SABA-only category (Fig. 1). An education process is paramount to inform patients, caregivers, physicians, and healthcare providers, such as pharmacists, that overuse of any reliever is not acceptable therapy, is potentially harmful, and needs further evaluation and management.

## Data Availability

Data sharing is not applicable to this article as no datasets were generated or analyzed during the current study.

## Abbreviations

CTS: Canadian Thoracic Society
ICS: Inhaled corticosteroid
LABA: Long-acting beta-agonist
PRN: As-needed
SABA: Short-acting beta-agonists
